# Flexible Radio-Frequency Identification (RFID) Tag Antenna for Sensor Applications

**DOI:** 10.3390/s18124212

**Published:** 2018-09-30

**Authors:** Mohammad Tariqul Islam, Touhidul Alam, Iskandar Yahya, Mengu Cho

**Affiliations:** 1 Centre of Advanced Electronic and Communication Engineering, Faculty of Engineering and Built Environment, Universiti Kebangsaan Malaysia, Bangi 43600, Selangor, Malaysia; touhid13@siswa.ukm.edu.my (T.A.), skandar.yahya@ukm.edu.my (I.Y.); 2 Laboratory of Spacecraft Environment Interaction Engineering (LaSEINE), Kyushu Institute of Technology, Fukuoka 804-8550, Japan; cho@ele.kyutech.ac.jp

**Keywords:** flexible RFID tag, long read range, RFID sensor ink-jet printing, UHF band

## Abstract

In this paper, an inkjet-printed flexible Radio-Frequency Identification (RFID) tag antenna is proposed for an ultra-high frequency (UHF) sensor application. The proposed tag antenna facilitates a system-level solution for low-cost and faster mass production of RFID passive tag antenna. The tag antenna consists of a modified meander line radiator with a semi-circular shaped feed network. The structure is printed on photo paper using silver nanoparticle conductive ink. The generic design outline, as well as tag antenna performances for several practical application aspects are investigated. The simulated and measured results verify the coverage of universal UHF RFID band with an omnidirectional radiation pattern and a long-read range of 15 ft. In addition, the read range for different bending angles and lifetimes of the tag antenna are also demonstrated.

## 1. Introduction

Nowadays, Radio Frequency Identification (RFID) technology is playing a promising role for new generation non-invasive RFID sensor applications. The tag antenna is a key element of the RFID system. The read range is highly dependent on the tag antenna itself. A flexible tag antenna with an omnidirectional radiation pattern is essential for smooth RFID sensor application in various fields, like child tracking in childcare centers, patient tracking in hospital management systems, internet of things (IoT) RFID sensors, epidermal sensors, etc. [1,2,3,4,5]. The major challenges that could potentially hinder the practical implementation of RFID are cost-effectiveness, reliability, and flexibility of materials. Several studies have been performed to address these challenges [6,7,8,9]. Inkjet printing technology is a promising technique for the low-cost fabrication of electronics and radio frequency (RF) circuit. Refs. [10,11,12,13] using engineered conductive inks made from silver nanoparticles, carbon nanotubes, or organometallic particles [14]. At the beginning this technology required thermal sintering at a high temperature, but now, due to developments in material science, new types of conductive ink have been developed which dry instantly at room temperature [11].

A temporary tattoo type flexible RFID antenna was presented for epidermal sensor application in [5], where the tag antenna was fabricated by profiling a silver painting technique. A maximum read range of 1.2 m was measured at 918 MHz. Besides this, a loop tag antenna was designed on Styrofoam to achieve a frequency range of 902–928 MHz (North America) [15]. Nonetheless, the antenna has a large size of 90 × 90 mm^2^ and the use of a near-air dielectric constant substrate may cause a change in antenna performance. A graphene-nanoflake-printed meandered line tag antenna is reported in [16], which achieved a maximum gain of −4 dBi and a radiation efficiency of 32%. The antenna was printed on flexible photo paper. In [17], a graphene-based flexible tag antenna is proposed for humidity-sensing applications. The tag antenna achieved a maximum read range of about 2 m with a size of 100 × 20 mm^2^.

A multi-layered tag antenna with a parasitic radiator was developed in [7] to achieve stable performance at the near field of the metallic object. Although the developed tag is quite a lot smaller in size, the structure is not flexible. Flexible polymeric substrates have been used to fabricate UHF RFID tag antenna in [8], but the maximum read range is below 3 m. Moreover, a double-T feeding-technique-based RFID tag antenna with an alphabetical pattern was proposed for long read range RFID application in [18]. The tag antenna was fabricated on hard FR4 substrate material and flexible polycarbonate substrate material with a size of 89.5 mm × 25 mm. 

Besides this, a new approach of 3D printed wearable RFID tag antenna has been developed, which achieved a maximum working distance of about 4.30 m [19]. Another approach that combines three-dimensional and inkjet printing technologies has been developed to reduce the tag antenna size (radius 15 mm, height 7.5 mm), and can cover a maximum reading range of 2.1 m [20]. In [21], a 3D-printed flexible RFID tag antenna is presented, the tag achieved a 10.6 m read range, although the read range decreased with increasing the number of stretching. The overall dimension of the tag antenna is 140 mm × 30 mm × 1.2 mm. 

In this paper, a passive printed tag antenna with an omnidirectional radiation pattern and a 15 ft (4.57 m) long measured read range is presented. The antenna encompasses the universal UHF RFID band (860–960 MHz). It exhibits a low-profile, compact, and flexible structure that makes it appropriate for RFID sensor applications. 

## 2. Design Methodology

**Figure 1:**
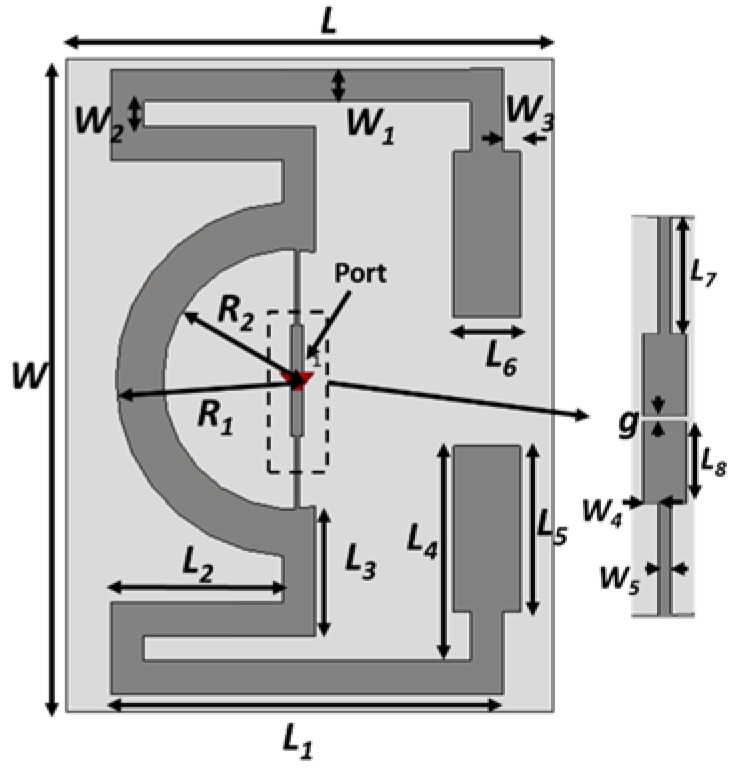
Design layout of the developed Flexible Radio-Frequency Identification (RFID) tag.

The design layout of the proposed RFID tag antenna is presented in Figure 1. The modified meander technique has been utilized to minimize the size of the tag antenna structure. The low-cost biodegradable paper substrate with a dimension of 44 × 59 × 0.54 mm^3^, a dielectric constant of 3.2, and a loss tangent of 0.05 is used to fabricate the RFID tag antenna. The use of a paper substrate makes the tag antenna flexible and opens a wide avenue for bending tag applications. The commercially available NXP SL3S1213 UCODE G2iL chip [22] is used to communicate with the reader end. A semi-circular loop with feed lines has been optimized to match with a chip impedance of 23-j224 Ω. Moreover, the meander line radiator is optimized in such a way that both arms keep balance, which helps to accomplish the donut-shaped radiation pattern and compliance with the dipole-type radiation pattern. The radiating element of the presented tag antenna is printed using silver nanoparticle ink. The commercially available DCP-T500W Inkjet printer has been utilized to print the tag antenna. The Refill Tanks of the DCP-T500W printer were filled by Ag-nanoparticle ink [23] using disposable syringe filters. The chip was connected to the antenna through the silver-based conductive glue. The optimized design parameter is listed in Table 1. 

## 3. Results and Discussion

**Figure 2:**
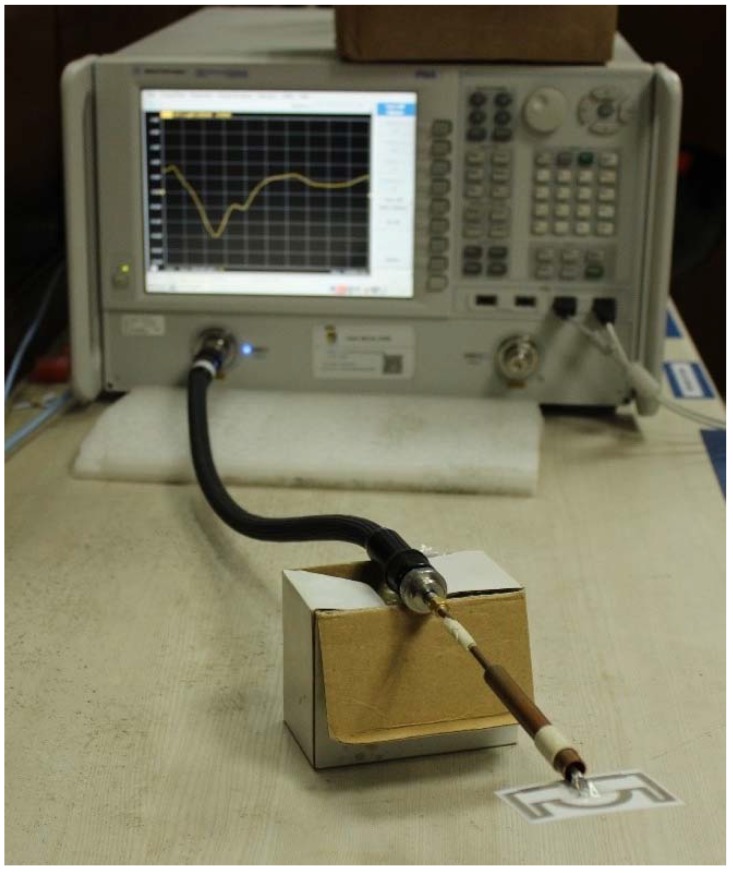
Reflection coefficient measurement of the fabricated prototype.

**Figure 3:**
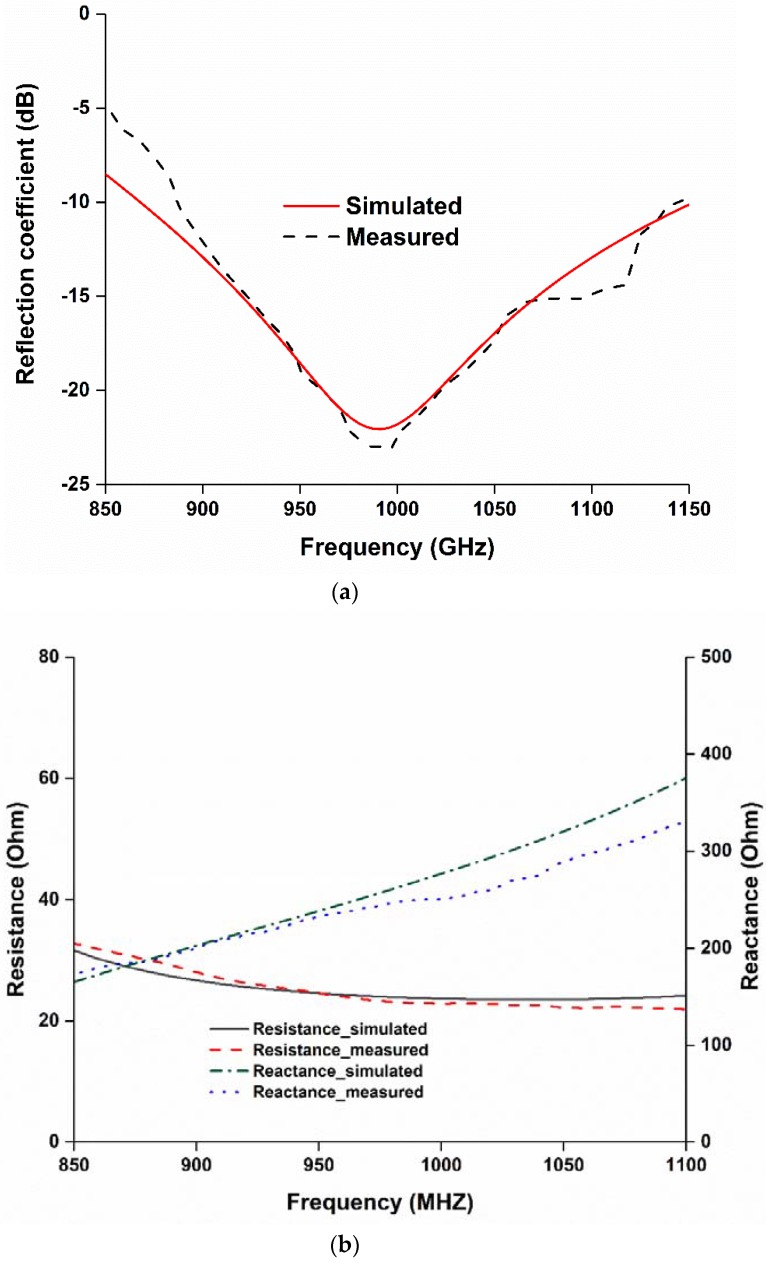
(**a**) Reflection coefficient and (**b**) input impedance of the proposed RFID tag.

The reflection coefficient of the fabricated tag antenna has been measured using a performance network analyzer (PNA) Agilent N5227A (Keysight Technologies, Santa Rosa, CA, United States), shown in Figure 2. The tag antenna is fed using a balun, which is λ/4 in length. The tag antenna shows a fractional impedance bandwidth (S11 < −10 dB) of 34.156% (0.855–1.155 GHz), which can operate within the Universal UHF RFID band as well as Malaysia RFID band (919 to 923 MHz). The measured and simulated results in Figure 3a show good agreement. However, a little mismatch is observed. This mismatch is possibly due to a printing error or a balun soldering error. The input impedance of the proposed tag antenna has been investigated to verify that it matches with the chip impedance, shown in Figure 3b.

**Figure 4:**
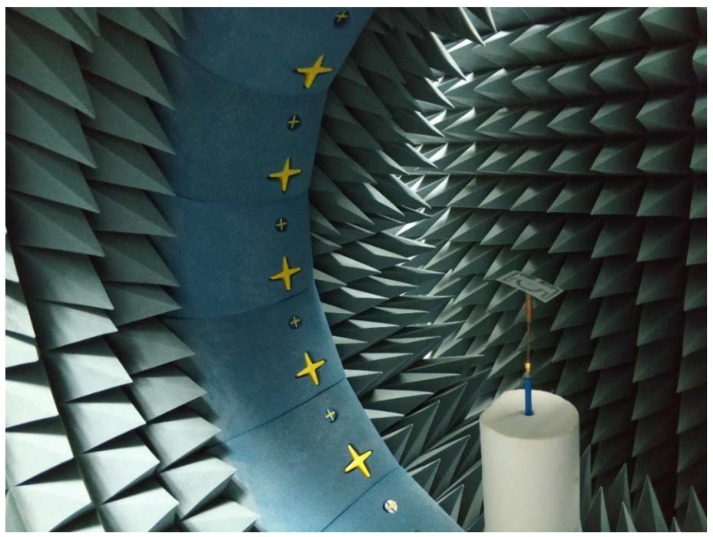
Far-field characteristics measurement using a Satimo near-field measurement system.

**Figure 5:**
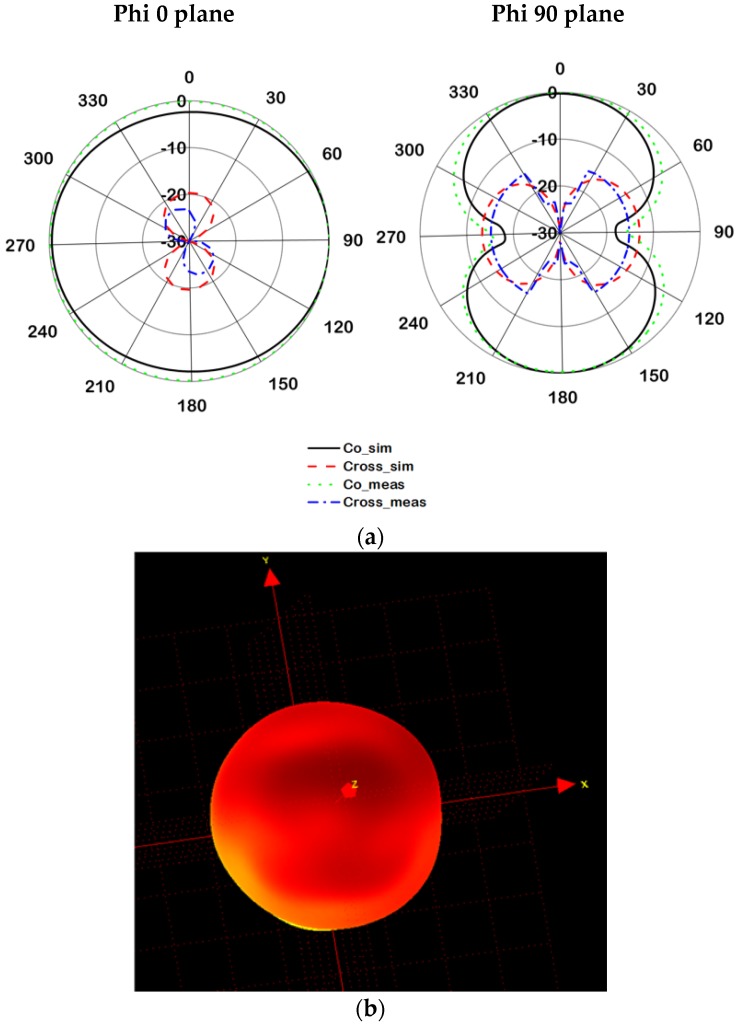
Radiation pattern at 921 MHz (**a**) 2D polar plot and (**b**) 3D pattern.

The far-field radiation characteristics of the proposed tag antenna have been measured using the Satimo near-field measurement system, shown in Figure 4. The radiation patterns for both phi 0 and 90° planes are demonstrated in Figure 5. The proposed tag antenna shows an omnidirectional radiation pattern at phi 0° plane and a donut-shaped pattern at the phi 90° plane. The balanced meander line radiator helps to accomplish the donut-shaped radiation pattern, which complies with the dipole-type radiation pattern. In addition, the 3D radiation pattern is depicted in Figure 5b to realize the real field scenario of the antenna radiation pattern.

**Figure 6:**
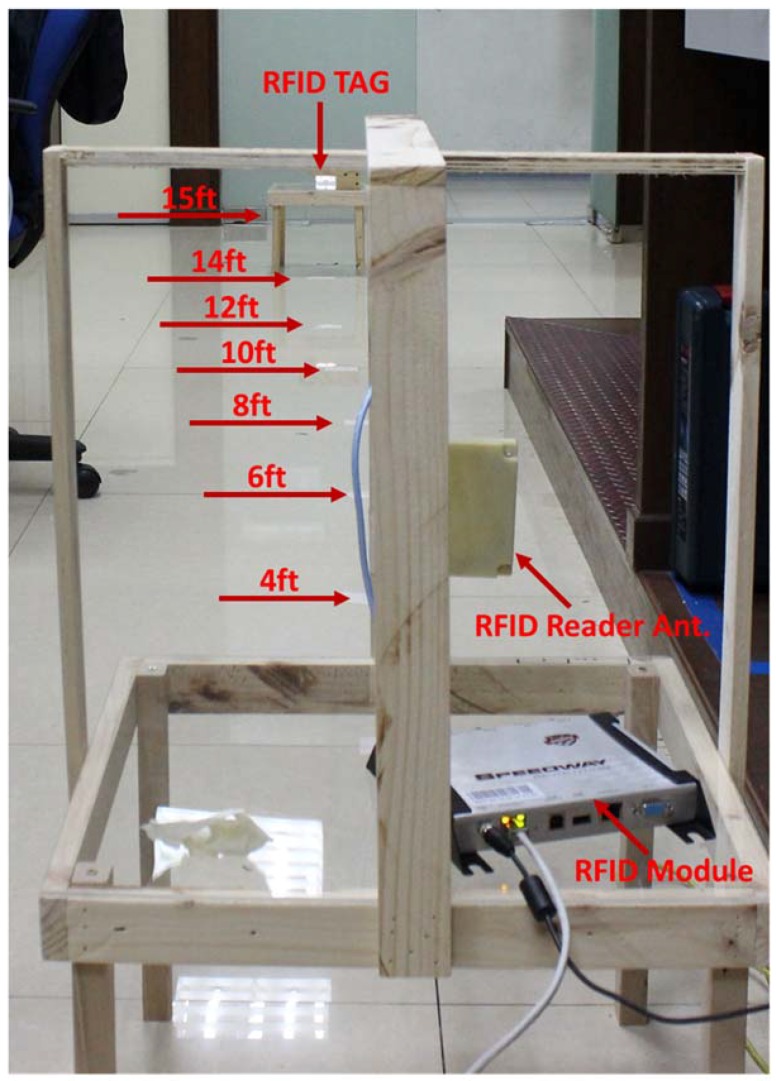
Read range measurement setup.

**Figure 7:**
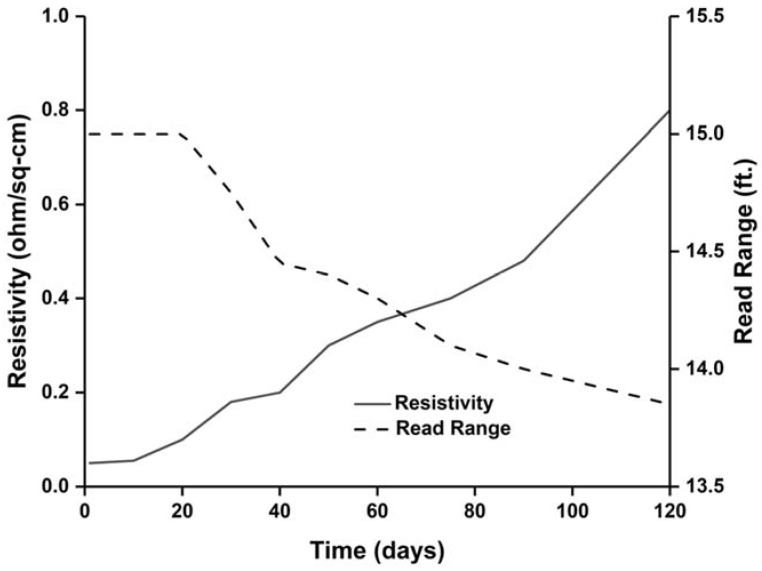
Resistivity and read range of the proposed tag antenna with respect to time.

**Figure 8:**
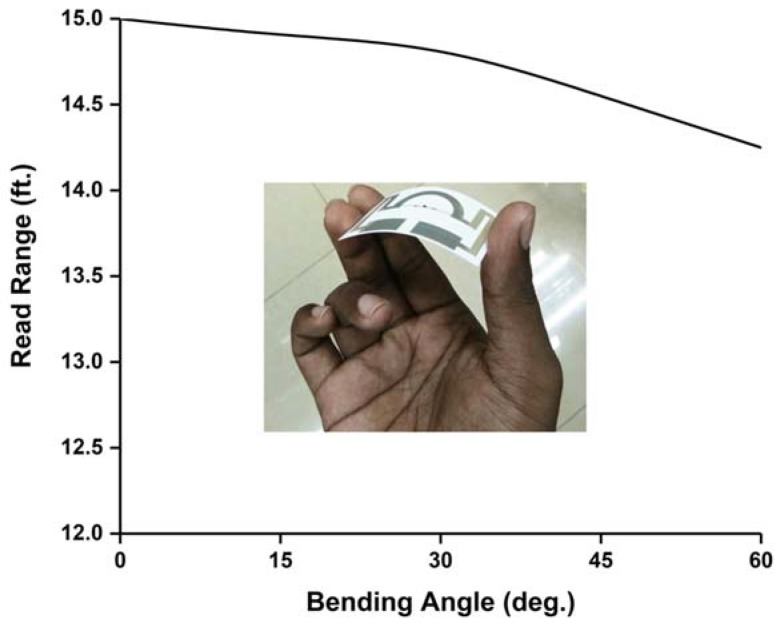
Read range analysis for different bending conditions.

Read range of the tag antenna is a very important parameter for RFID application. The measured read range pattern presents a fundamental criterion of the real scenario antenna radiation. The read range measurement setup is depicted in Figure 6. The measurement setup has been arranged using an Impinj Speedway revolution R420 UHF RFID reader module and a circularly polarized patch antenna with a gain of 3.4 dBi. The NXP IC chip comprising a sensitivity of −18 dBm. The developed RFID tag depicts a long-read range of approximately 15 ft (4.57 m). Although Circular Polarization (CP) has less read range compared to Linear Polarization (LP) for the same amount of gain and power, if an LP reader antenna is used to read the developed antenna, the tag antenna can read a longer distance. In addition, the antenna life performance has been investigated and is depicted in Figure 7. The test has been conducted for 120 days. The resistivity and read range have been measured every 15 days during this period. It is observed from Figure 7 that the resistivity increases with respect to time and the read range decreases accordingly. Besides this, the read range performance has been investigated for different bending conditions, as illustrated in Figure 8. The proposed tag antenna shows a stable performance for a bending condition of 0 to 60°. Comparisons of tag antenna performance with recent works have been depicted in Table 2, which shows that the proposed tag antenna achieves a good read range with respect to tabulated works.

## 4. Conclusions

This paper presents a paper-based flexible UHF RFID tag antenna for sensor applications. The design used a semi-circular shaped feed network with a meander line radiating element, printed using inkjet printing technology. The antenna is printed on photo paper using silver nanoparticle conductive ink. The proposed tag antenna offers eco-friendly low-cost RFID tag service for sensor modules. The read range of the proposed antenna has been validated using an RFID reader module and a read range of about 4.57 m is found when 2.0 W Effective radiated power (ERP) power is applied to the reader antenna. Moreover, the flexibility and antenna lifetime performance have also been studied and displayed a stable performance.

## Figures and Tables

**Table 1:** Optimized tag antenna design parameters.

Parameters	Value (mm)	Parameters	Value (mm)
*L*	44	*L*_8_	4.83
*W*	59	*R*_1_	16
*L*_1_	32.5	*R*_2_	11.75
*L*_2_	15.5	*g*	0.65
*L*_3_	11.75	*W*_1_	3.25
*L*_4_	22.5	*W*_2_	2.28
*L*_5_	15	*W*_3_	1.5
*L*_6_	6	*W*_4_	0.3
*L*_7_	6.82	*W*_5_	0.7

**Table 2:** Comparison with some existing works.

Ref.	Tag Antenna Dimension (mm)	Antenna Type	Antenna Gain (dB)	Antenna Materials	Fabrication Technique	Read Range (m)
[5]	65 × 20	flexible	−15.5	inkjet paper	tattoo transfer	1.2
[8]	135.7 × 22.2	Not flexible	1.021	FR4	screen printing	2.8
[24]	96 × 50 × 2	not flexible	-	FR4	etching	4
[25]	100 × 20	flexible	−2.18	cardboard	doctor-blading	4.5
[26]	30 × 30 × 3	flexible	−3	polyethylene terephthalate	deposition	3.5
Proposed	44 × 59	flexible	2.12	photo paper	inkjet printing	4.57
